# Metagenomes from Soils along an Agricultural Transect in Ulster County, New York

**DOI:** 10.1128/mra.01015-22

**Published:** 2023-02-13

**Authors:** Carolina Oliveira de Santana, Pieter Spealman, David Gresham, Gabriel G. Perron

**Affiliations:** a Bard Center for Environmental Sciences and Humanities, Bard College, Annandale-on-Hudson, New York, USA; b Center for Genomics and Systems Biology, New York University, New York, New York, USA; c Department of Biology, Reem-Kayden Center for Science and Computation, Bard College, Annandale-on-Hudson, New York, USA; University of Strathclyde

## Abstract

Many modern farming practices negatively impact ecosystems on the local and global scales. Here, we assessed the taxonomic structures of 48 soil microbial communities along an agricultural transect using 16S rRNA and internal transcribed spacer (ITS) amplicon sequencing. We further characterized the functional structures of a subsample of 12 microbiomes using whole-genome sequencing.

## ANNOUNCEMENT

Microbial communities in agricultural soil play important ecological and economic roles (1, 2). Many modern agricultural practices negatively impact soils and soil microbiomes, degrading nutrients and reducing diversity (3). The use of antimicrobials in agriculture further alters microbial communities in soil and surrounding environments (4). Given increasing pressures on the global food system, understanding how soil microbiomes respond to agricultural practices is critical. Here, we present metagenomic sequences from soil samples collected from an agricultural transect encompassing different proximities to human activity.

**FIG 1:**
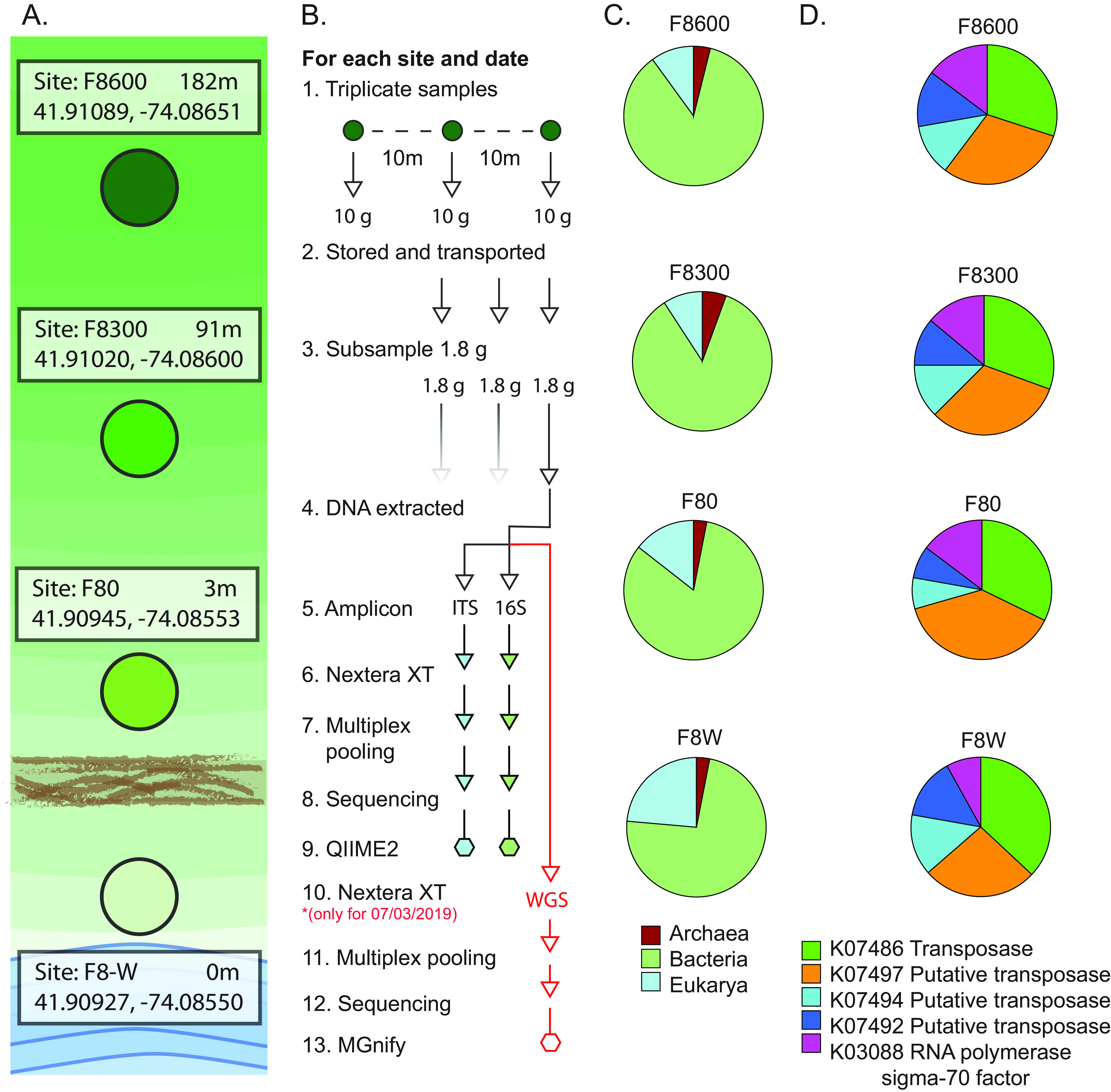
(A) Schematic diagram showing the distance from the water line (white circle) of each site along the F8 transect from which triplicate samples were taken in 2019. (B) Schematic diagram of the sampling, library construction, and sequencing of each sample. (C) Pie charts showing the relative proportions of eukaryotes, prokaryotes, and archaea. (D) Percent abundances of the top five most abundant KO orthologs.

Samples were collected from four sites along a transect of previously farmed soil, perpendicular to Esopus Creek, New York (Fig. 1A), in a gradient from most affected by human activity to least, as follows: F8-W, a forested strip between the riverbank and a dirt road; F8-0 (3.05 m), F8-300 (91.44 m), and F8-600 (182.88 m), fallow farm sites. At each site, we collected three 10-g topsoil samples on 6 June, 19 June, and 3 July 2019 using sterile techniques. Samples were transported to the laboratory on ice in a dark cooler and frozen for at least 24 h.

For each sample (Fig. 1B), we extracted DNA from 1.8 g of soil using the Quick-DNA fecal/soil microbe miniprep kit (Zymo), and this DNA was used for 16S rRNA and internal transcribed spacer (ITS) amplicon sequencing; for samples collected on 3 July, these extractions were also used for whole-genome sequencing (WGS). All 16S rRNA amplicon sequencing amplified the V4 region using the 515F and 806R primers (5). ITS amplicon sequencing used the ITS1f and ITS2 primers (5). PCR products for the 16S rRNA and ITS amplicons were pooled separately and purified on a 2% agarose gel using a Qiagen gel extraction kit. The excised regions were ~385 bp for the 16S rRNA amplicons and 200 to 600 bp for the ITS amplicons. WGS library preparation was performed using the Nextera XT DNA library preparation kit (Illumina). All purified libraries were quality checked using an Agilent 2100 BioAnalyzer and a high-sensitivity DNA kit (Agilent) and stored at −20°C until sequencing. Amplicon libraries were sequenced at Wright Labs (Huntingdon, PA) using Illumina MiSeq v2 paired-end sequencing (2 × 250-bp reads) with 20% PhiX spike-in. WGS libraries were sequenced using an Illumina NextSeq 2000 system (2 × 150-bp paired-end reads) with default parameters.

For 16S rRNA and ITS analyses, the QIIME2 pipeline was used with default parameters except for DADA2 (16S rRNA, denoise-paired, –p-trim-left-f 0 –p-trim-left-r 0 –p-trunc-len-f 250 –p-trunc-len-r 250; ITS, denoised-single, –p-trunc-len 150). For WGS, raw-read processing, assembly, and analysis were performed using the MGnify v5.0 pipeline (6) with default parameters for adapter trimming, quality filtering, and subsequent analysis. The results for each sample are shown in Table 1. We found that the majority of small subunit (SSU)- and large subunit (LSU)-containing contigs identified by MGnify belonged to bacteria (82%), eukarya (14%), and archaea (4%) (Fig. 1C). From 149,688 contigs, MGnify identified 208,556 coding sequences associated with 6,039 genome properties (7). Transposases and putative transposases (0.8% of KEGG Orthology [KO] orthologs) accounted for the majority of high-abundance KO orthologs in each sample (11.5% of all KO orthologs assigned) (Fig. 1D).

**TABLE 1:** Summary of data

Sample^*a*^	SRA or MGnify accession no.	Mean read length (bp)	Total no. of read pairs submitted	Total no. of read pairs	Total no. of contigs	*N*_50_ (bp)
WGS						
F8W_T25	ERR9752702	151	13,493,283			
F8W_T26	ERR9752724	151	11,497,693			
F8W_T27	ERR9752734	151	9,693,650			
F80_T28	ERR9752742	151	9,510,055			
F80_T29	ERR9752750	151	11,153,342			
F80_T30	ERR9752757	151	10,188,435			
F8300_T31	ERR9752762	151	13,358,533			
F8300_T32	ERR9752768	151	12,361,583			
F8300_T33	ERR9752775	151	11,262,599			
F8600_T34	ERR9752780	151	11,369,775			
F8600_T35	ERR9752794	151	14,442,272			
F8600_T36	ERR9760450	151	16,556,960			
ITS						
F80_1_0607	ERR10168568	151	1,444,523			
F80_1_0619	ERR10168570	151	1,444,589			
F80_1_0703	ERR10168632	151	2,185,005			
F80_2_0607	ERR10168636	151	2,328,428			
F80_2_0619	ERR10168647	151	1,453,311			
F80_2_0703	ERR10168649	151	1,011,053			
F80_3_0607	ERR10168652	151	948,188			
F80_3_0619	ERR10168654	151	1,571,348			
F80_3_0703	ERR10168656	151	975,492			
F8300_1_0607	ERR10168659	151	1,095,855			
F8300_1_0619	ERR10168660	151	787,551			
F8300_1_0703	ERR10168662	151	1,072,100			
F8300_2_0607	ERR10168666	151	1,319,891			
F8300_2_0619	ERR10168672	151	1,446,988			
F8300_2_0703	ERR10168675	151	1,152,882			
F8300_3_0607	ERR10168677	151	1,414,719			
F8300_3_0619	ERR10168680	151	23			
F8300_3_0703	ERR10168682	151	1,489,768			
F8600_1_0607	ERR10168684	151	1,456,243			
F8600_1_0619	ERR10168688	151	3,790,963			
F8600_1_0703	ERR10168745	151	1,336,475			
F8600_2_0607	ERR10168750	151	1,154,444			
F8600_2_0619	ERR10168753	151	506,739			
F8600_2_0703	ERR10168760	151	1,315,871			
F8600_3_0607	ERR10168776	151	1,582,067			
F8600_3_0619	ERR10168779	151	1,398,289			
F8600_3_0703	ERR10639905	151	47			
F8W_1_0607	ERR10213569	151	2,199,554			
F8W_1_0619	ERR10213571	151	873,471			
F8W_1_0703	ERR10213561	151	1,256,782			
F8W_2_0607	ERR10213574	151	656,150			
F8W_2_0619	ERR10213577	151	1,178,439			
F8W_2_0703	ERR10213582	151	759,588			
F8W_3_0607	ERR10213585	151	1,327,567			
F8W_3_0619	ERR10213587	151	1,281,001			
F8W_3_0703	ERR10213589	151	926,657			
16S rRNA						
F80_1_2019_06_07_16S	ERR10169571	250.7	30,535			
F80_1_2019_06_19_16S	ERR10169574	250.6	36,286			
F80_1_2019_07_03_16S	ERR10169578	250.6	21,911			
F80_2_2019_06_07_16S	ERR10169580	250.7	7,794			
F80_2_2019_06_19_16S	ERR10169582	250.6	24,368			
F80_2_2019_07_03_16S	ERR10169585	250.6	18,269			
F80_3_2019_06_07_16S	ERR10169587	250.6	30,048			
F80_3_2019_06_19_16S	ERR10169589	250.6	34,117			
F80_3_2019_07_03_16S	ERR10169592	250.6	20,410			
F8300_1_2019_06_07_16S	ERR10169803	250.6	33,208			
F8300_1_2019_06_19_16S	ERR10169804	250.6	44,023			
F8300_1_2019_07_03_16S	ERR10169815	250.6	23,870			
F8300_2_2019_06_07_16S	ERR10169821	250.6	28,959			
F8300_2_2019_06_19_16S	ERR10169825	250.5	36,641			
F8300_2_2019_07_03_16S	ERR10169841	250.7	17,673			
F8300_3_2019_06_07_16S	ERR10169845	250.6	27,000			
F8300_3_2019_06_19_16S	ERR10169847	250.6	38,461			
F8300_3_2019_07_03_16S	ERR10169849	250.6	30,309			
F8600_1_2019_06_07_16S	ERR10169850	250.6	28,737			
F8600_1_2019_06_19_16S	ERR10169852	250.6	21,212			
F8600_1_2019_07_03_16S	ERR10169854	250.6	42,005			
F8600_2_2019_06_07_16S	ERR10169855	250.7	29,635			
F8600_2_2019_06_19_16S	ERR10169856	250.6	34,151			
F8600_2_2019_07_03_16S	ERR10169859	250.6	40,674			
F8600_3_2019_06_07_16S	ERR10169861	250.6	50,432			
F8600_3_2019_06_19_16S	ERR10169885	250.6	37,360			
F8600_3_2019_07_03_16S	ERR10169864	250.7	33,704			
F8W_1_0607_16S	ERR10213607	250.6	46,515			
F8W_1_0619_16S	ERR10213609	250.6	48,041			
F8W_1_0703_16S	ERR10213610	250.6	16,155			
F8W_2_0607_16S	ERR10213612	250.4	288			
F8W_2_0619_16S	ERR10213613	250.6	44,701			
F8W_2_0703_16S	ERR10213614	250.7	15,809			
F8W_3_0607_16S	ERR10213616	250.6	34,981			
F8W_3_0619_16S	ERR10213618	250.6	54,720			
F8W_3_0703_16S	ERR10213620	250.6	39,692			
MGnify						
F80_T28	MGYA00607100			9,016,418	9,977	562
F80_T29	MGYA00607095			10,558,938	833	560
F80_T30	MGYA00607099			9,646,580	7,393	554
F8300_T31	MGYA00607093			12,653,552	17,047	566
F8300_T32	MGYA00606633			11,703,364	14,294	555
F8300_T33	MGYA00607091			10,659,098	10,304	555
F8600_T34	MGYA00607098			10,767,204	11,398	558
F8600_T35	MGYA00607096			13,663,903	13,799	557
F8600_T36	MGYA00607092			15,683,527	20,013	564
F8W_T25	MGYA00607094			12,781,962	22,814	572
F8W_T26	MGYA00607097			10,887,794	7,033	551
F8W_T27	MGYA00607090			9,183,848	7,283	556

a Sequencing run statistics are available for WGS, ITS amplicon sequencing, and 16S rRNA V4 amplicon sequencing, including the sample name (for WGS, site_id; for ITS, site_replicate_date; for 16S, site_replicate_date_16S), read accession number, mean read length, and total reads or read pairs submitted. WGS raw reads were used to perform metagenomic analyses using MGnify, which are accessible using the MGnify accession numbers.

### Data availability.

All data are available through EMBL/EBI BioProject accession number PRJEB52998 and the MGnify identification number MGYS00006044. Individual accession numbers for each sample are available on Table 1.
